# Anatomical characteristics of the styloid process associated with internal carotid artery dissection: a systematic review and meta-analysis of controlled trials

**DOI:** 10.3389/fradi.2026.1788985

**Published:** 2026-05-01

**Authors:** Tomas Klail, Emmanouil Kalioras, Marc von Gernler, Roland Giger, Aristomenis K. Exadaktylos, Martin Müller, Franca Wagner

**Affiliations:** 1 University Institute of Diagnostic and Interventional Neuroradiology, Inselspital, Bern University Hospital, Faculty of Medicine, University of Bern, Switzerland; 2 Institute of Radiology, Cantonal Hospital Münsterlingen, Münsterlingen, Switzerland; 3 Medical Library, University Library of Bern, University of Bern, Bern, Switzerland; 4 Department of Otorhinolaryngology, Head and Neck Surgery, Inselspital, Bern University Hospital, Faculty of Medicine, University of Bern, Switzerland; 5 Department of Emergency Medicine, Inselspital, Bern University Hospital, Faculty of Medicine, University of Bern, Switzerland; 6 Department of Diagnostic and Interventional Neuroradiology, Cantonal Hospital Aarau, Aarau, Switzerland

**Keywords:** anatomical risk factors, dissection, eagle syndrome, internal carotid artery, styloid process

## Abstract

**Purpose:**

Internal carotid artery dissection (ICA-D) is an important cause of stroke in adults. The styloid process (SP) may be associated with ICA-D due to potential (micro)trauma resulting from its close anatomical proximity to the internal carotid artery (ICA). The aim of this systematic review with meta-analysis is to investigate the association between SP characteristics –particularly SP-ICA distance – and ICA-D.

**Methods:**

A systematic review was conducted across six databases to identify observational studies comparing ICA-D patients to controls. The primary outcome of interest was the association between SP-ICA distance and ICA-D. Secondary outcomes included associations between ICA-D and the SP length or angulation. A random-effects meta-analysis was performed, including a subgroup analysis of moderate/high-quality studies. Effect sizes were expressed as standardized mean differences (SMD, Hedges' g).

**Results:**

Six studies were included in the systematic review, of which five were eligible in the meta-analysis. The pooled analysis of all five case-control studies (270 ICA-D patients and 377 controls) showed no significant difference in SP-ICA distance (SMD = −0.92, *p* = 0.143); with a high degree of heterogeneity (I^2^ = 98%). Subgroup analysis of moderate/high-quality studies evaluating the SP-ICA distance ipsilateral to the ICA-D (4 studies) yielded a negative pooled SMD (−0.29, *p* = 0.047; moderate heterogeneity: I^2^ = 64%), consistent with a shorter SP-ICA distance in ICA-D cases. Meta-analysis of the SP length (3 studies) found no significant association (SMD 0.24, *p* = 0.139) and two studies also found no significant relationship between ICA-D and SP angulation.

**Conclusion:**

A shorter SP–ICA distance was associated with ICA-D, whereas no significant associations were observed for SP length or angulation. However, the available evidence remains limited and heterogeneous.

**Systematic Review Registration:**

CRD42024582594

## Introduction

Internal carotid artery dissection (ICA-D) accounts for approximately 20% of ischemic strokes in adults under 50 years of age (1). Several risk factors – including connective tissue disorders, infections, and minor trauma – have been associated with ICA-D (2, 3); however, no definitive causal mechanisms have been established, except in cases linked to recent trauma (4). The underlying pathophysiology remains incompletely understood (5) and the majority of cases (∼60%) are classified as spontaneous (6).

The styloid process (SP), a bony projection of the temporal bone, has gained attention as a potential anatomical risk factor due to its possible impingement on the internal carotid artery (ICA), resulting in a vascular variant of Eagle syndrome (7, 8). Traditionally, compression of the ICA by an elongated SP has been linked to arterial stenosis and ischemic symptoms. More recently, the potential role of the SP in ICA-D has been explored. Repetitive microtrauma caused by the SP may lead to irritation of the ICA wall and increase the risk of dissection (9).

Despite this emerging evidence, only one meta-analysis has synthesized the evidence, focusing exclusively on SP length as a potential risk factor for ICA-D (10). This study reported a tendency for patients with ICA-D to have a longer SP; however, methodological limitations – including a narrow search strategy, limited database selection, and the exclusion of other anatomical factors – constrain the generalizability of the findings. Importantly, SP-ICA distance was not evaluated, although it may represent be a more relevant determinant of mechanical interaction than SP length alone. Given the considerable anatomical variability of the ICA course and inconsistent proximity to the SP (11), we hypothesized that SP–ICA distance differs between patients with and without ICA-D and is more relevant than SP length or angulation. The aim of this systematic review and meta-analysis was to assess the association between styloid process characteristics and ICA-D, with a particular focus on SP–ICA distance.

## Methods

This systematic review with meta-analysis was conducted in accordance with the Preferred Reporting Items for Systematic reviews and Meta-Analyses (PRISMA) guideline (12). Ethics approval or patient consent were not required, as the study was based exclusively on published data. The protocol was registered at the PROSPERO database (registration number: CRD42024582594) (13).

### Eligibility criteria

This systematic review included observational studies assessing the occurrence of ICA-D in relation to anatomical characteristics of the SP compared with controls without ICA-D. Eligible studies were required to use computed tomography angiography (CTA), magnetic resonance angiography (MRA), interventional angiography, or pathological examination to evaluate the ICA, SP, and their spatial relationship. Studies using ultrasound were excluded due to operator dependence and limited reliability (14), as were studies with inaccessible full texts. Studies were included in the meta-analysis if they provided sufficient data for quantitative synthesis, such as mean or median values with standard deviation (SD), confidence interval (CI), or interquartile range in both groups, or reported effect measures (e.g., standardized mean difference [SMD] or odds ratio [OR] with 95% CI) for the outcome of interest.

### Search strategy

A comprehensive literature search was developed and conducted by a medical information specialist (MG) across the following databases:

- Medline (Ovid) (Ovid Medline(R) all, 1946 to 11 July 2024)
- Embase (Ovid) (1974 to 11 July 2024)
- Cinahl (EBSCO) (Cinahl with full text, 1963 to 11 July 2024)
- Cochrane Library (Wiley) (1996 to 11 July 2024)
- Scopus (Elsevier) (1970 to 11 July 2024)
- Web of Science Core Collection (1990 to 11 July 2024)

The search strategy was based on the key concepts “styloid process” and “carotid artery dissection.” No restrictions were applied regarding study design, language, publication year, or other formal criteria. All searches were performed on 11 July 2024. The full search strategy for all databases was published in searchRxiv after the initial search and prior to data extraction (Supplementary Methods 1). To enhance completeness, additional records were identified through Google Scholar (first 200 results) and by screening the reference lists of included studies.

### Potential anatomical SP characteristics as risk factors for ICA-D

**Figure 1:**
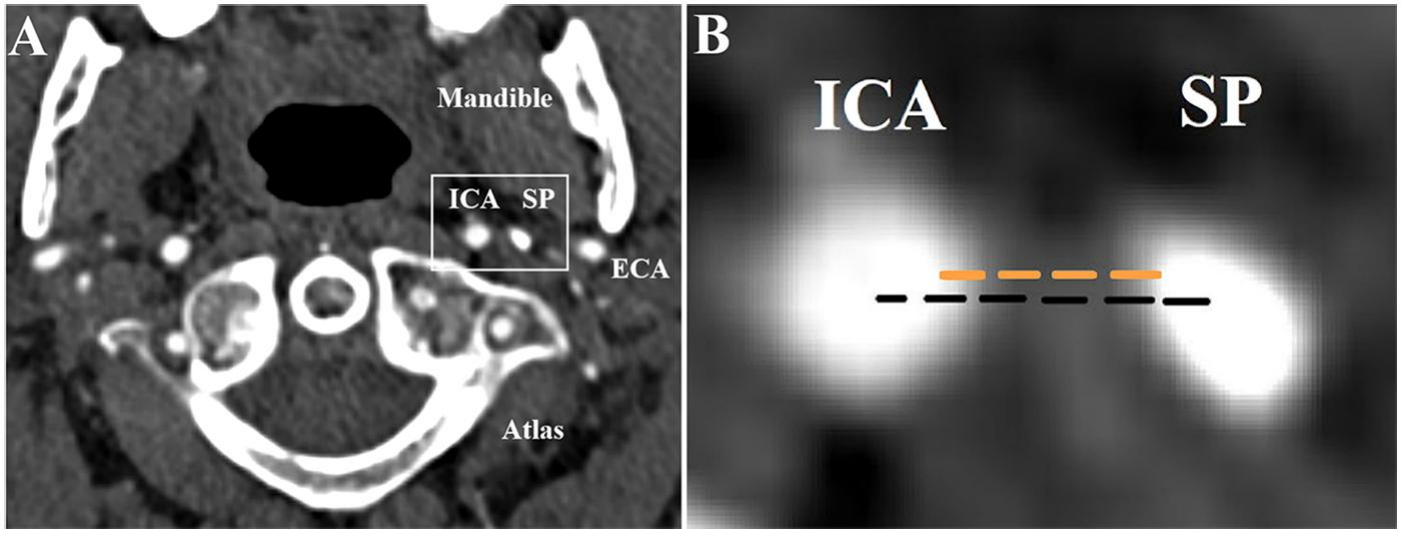
**A**. Axial CTA images and a detailed view of a healthy control subject, displaying the general anatomy on the axial view. **B**. Enlarged section marked with a white rectangle on image A, showing different approaches used in SP-ICA measurement (the shortest marginal SP-ICA distance is marked with orange dotted lines, the shortest intercentric SP-ICA distance with black dotted lines) CTA, computed tomography angiography; ICA, internal carotid artery; SP, styloid process; ECA, external carotid artery.

**Figure 2:**
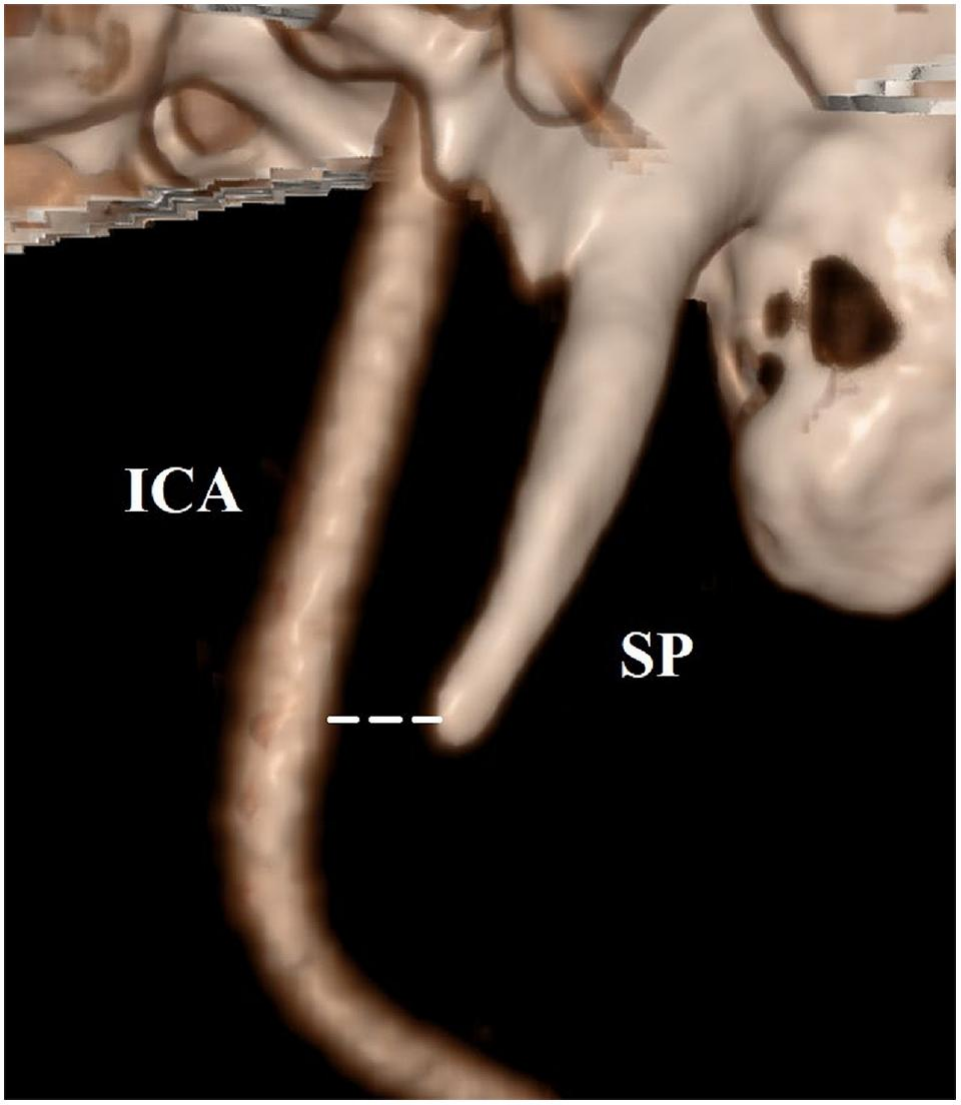
Measurement of the marginal shortest SP-ICA distance using axial view on 3D reconstruction of the CTA (the shortest marginal SP-ICA distance is marked with white dotted lines). CTA, computed tomography angiography; ICA, internal carotid artery; SP, styloid process.

The primary association of interest was the relationship between SP-ICA distance and ICA-D. Several methods have been used to measure SP–ICA distance. For this systematic review, *intercentric* SP-ICA distance was defined as the shortest distance between the center of the SP and the ICA. *Marginal* SP-ICA distance was defined as the shortest distance between the outer edge of the ICA vessel wall and the surface of the SP on axial imaging (Figures 1, 2). All other measurement approaches were categorized as “other methods” and are described in detail in study characteristics. Measurements performed ipsilateral and contralateral to the dissection were considered; when available, ipsilateral measurements were prioritized for the primary analysis.

Secondary associations of interest included SP length, measured from the skull base to the tip, and SP angulation, defined as medial angulation measured on the frontal plane or anterior angulation measured on the sagittal plane.

### Screening process

All identified records were imported into Covidence for study selection and data extraction (15). Duplicate records were removed automatically. Two independent reviewers (TK – 2 years of experience in neuroradiology, and EK – 5 years of experience in general radiology) screened and selected the studies. Discrepancies were resolved by a senior researcher (FW – 14 years of experience in neuroradiology), a board-certified neuroradiologist holding the European Diploma in Head and Neck Radiology.

### Data extraction

Data from the included studies were extracted using a standardized approach. The following study characteristics were recorded:

1. **Study Characteristics:** Publication year, study design (e.g., case-control, cohort, cross-sectional), matching or adjustment procedures (e.g., age, sex and vascular risk factors), exclusion of bilateral dissections, number of raters, and rater blinding.
2. **Case and control definitions:** Criteria used to define case and control groups, including exclusion of traumatic ICA dissections, information on the presence of high-grade ICA stenosis or complete ICA occlusion in cases.
3. **Population characteristics:** Mean age and sex distribution (number and percentage of male participants), as well as total sample size in cases and controls.
4. **Imaging and measurement approaches:** Imaging modalities (including MRI, where applicable), methods for measuring SP length, and approaches for assessing SP–ICA distance (e.g., intercentric vs. marginal), the vessel wall definition used for measurements (16), and data required to quantify the primary and secondary associations.
5. **Risk of bias assessment:** See below.

### Risk of bias assessment

The risk of bias in individual studies was assessed using the Newcastle-Ottawa Scale (NOS) by two independent reviewers (TK, EK) (17). Discrepancies were resolved by a third reviewer (MM) when consensus could not be reached.

Studies were evaluated across three domains: (1) selection of cases and controls (case definition, representativeness of cases, selection and definition of controls); (2) comparability of cases and controls based on study design or statistical adjustment; and (3) ascertainment of exposure and comparability of exposure assessment between cases and controls. Each study could receive up to one star per item in the selection and exposure domains and up to two stars for comparability, yielding a maximum of nine stars. The NOS item on non-response rate was not applicable to the included study designs and was excluded. Accordingly, all studies received one additional star for this domain. Studies with a score below 7 were considered low quality.

### Strategy for data synthesis

All statistical analyses were conducted using Stata 18.1 (StataCorp, The College Station, Texas, USA). A random-effects meta-analysis was performed to pool standardized mean differences (SMD, Hedges' g) with 95% confidence intervals for continuous variables, including SP–ICA distance and SP length. For studies reporting alternative effect measures (e.g., OR based on dichotomized SP–ICA distance), results were included only when they reflected a comparable definition and direction of the association (e.g., shorter SP–ICA distance associated with higher odds of ICA-D). These results were not pooled with SMDs but are presented separately. Other anatomical characteristics, such as SP angulation, were analyzed descriptively due to heterogeneity in measurement methods. Confounder-adjusted effect sizes were preferred over unadjusted values for meta-analysis. Missing SDs were derived using formulas from the Cochrane Handbook (Version 6.5) (18) and Tukey et al. (19) when means with CIs or medians with interquartile ranges were reported. Authors were contacted twice for missing data, and studies were excluded from meta-analysis if data remained unavailable.

Moderate- to high-quality studies were analyzed in a stratified analysis. Publication bias for SP–ICA distance was assessed using funnel plot inspection and Egger's regression in the subgroup of moderate/high-quality studies with low to moderate heterogeneity (I^2^ ≤ 75%). Heterogeneity was assessed using the I^2^ statistic (significant heterogeneity was defined as I^2^ > 75%). The studies were characterized based on methodological features (e.g., study design, matching procedures, blinding, and exclusion criteria) and sample characteristics (e.g., number of raters, total sample size, proportion of males and mean age). Forest plots were generated for each meta-analysis. Statistical significance was set at *p* < 0.05.

## Results

### Study selection

A total of 3,155 studies were identified through the database and register searches, and an additional 40 records were found during citation search of the included studies. After removal of 1,273 duplicates, 1,922 articles remained for screening. Following application of the inclusion and exclusion criteria, 1,907 studies were excluded. The remaining 15 studies underwent full-text assessment, of which nine were excluded due to an incorrect study focus (*n* = 5) (20–24), or an inappropriate study design (*n* = 4) (10, 25–27) (Supplementary Table 1).

**Figure 3:**
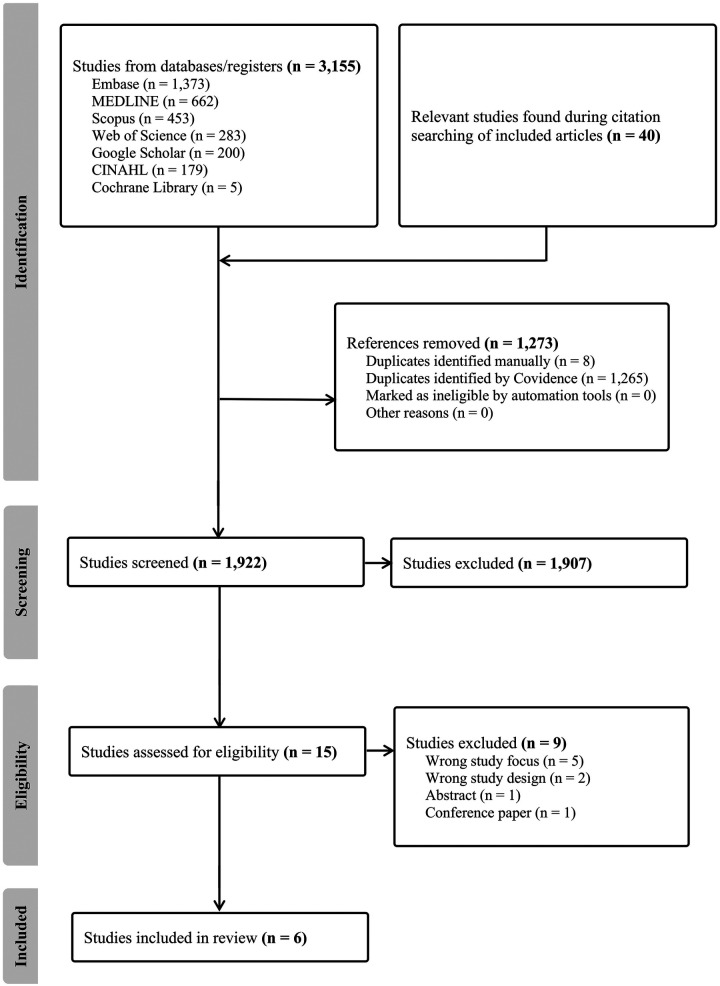
PRISMA flow chart.

Ultimately, six studies were included in the systematic review. No additional studies were identified through reference screening. For one study (28), unpublished data were obtained from the authors to supplement the published results. One study (11) was excluded from the meta-analysis due to missing SD data. The PRISMA flowchart illustrates the study selection process (Figure 3).

### Risk of bias among individual studies

One study scored 8 stars on the NOS, four studies scored 7 stars and one study scored 6 stars. All studies had a risk of bias in the control selection, as the CTA was performed only in clinically indicated cases and no studies had therefore healthy controls. Controls were selected from patients who underwent CTA and showed no evidence of ICA-D. Individual NOS scores are presented in Supplementary Table 2. The study by Venturini et al. declared open access funding by Università degli Studi di Firenze within the CRUI-CARE Agreement (9). No other included study declared any public, private or non-profit funding for their research.

### Study characteristics

In addition to NOS assessment, relevant baseline study characteristics are presented in Tables 1, 2.

**Table 1:** Baseline characteristics of the included studies.

Study (Year)	Design	Matching	Inclusion criteria	Exclusion criteria	Traumatic dissection excluded	Bilateral dissection excluded	Use of MRI*	No Raters	Blinding	Control definition	Diagnostic criteria for dissection presented
Venturini et al. (2023) (9)	Retrospective case-control study	Sex-matching	Acute cerebrovascular events who underwent CTA of the ICA	Not reported	No	No	No	2	No	Patients without ICA-D	No
Cruddas et al. (2021) (30)	Retrospective case-control study	Age- and sex-matched	CTA and positive for ICA-D	Iatrogenic ICA-D	No	No	No	1	No	Patients without evidence of ICA-D	No
Amorim et al. (2018) (29)	Retrospective case-control study	Age- and sex-matched	ICAD with IS, TIA or local symptoms and signs, with available CTA within 2 weeks after diagnosis	Not reported	No	No	No	2	No	Patients with ischemic stroke or TIA, caused by any etiology excluding ICA-D	No
Renard et al. (2013) (28)	Retrospective case-control study	Age- and sex-matched	Unilateral ICA-D	FMD, PA of ICA-D, CCA-D, recent history of trauma, neck surgery and aortic dissection	Yes	Yes	No	1	Yes	Patients without ICA-D and history of stenting or surgery of the ICA	No
Muthusami et al. (2013) (31)	Prospective case-control study	Age- and sex-matched	CTA and defined ICA-D criteria	Atherosclerotic or embolic origin, prior trauma, surgery and irradiation	Yes	No	No	2	Yes	Patients with stroke or TIA presentations that underwent CTA and did not have ICA-D	Yes
Raser et al. (2011) (11)	Retrospective case-control study	Age- and sex-matched	Unilateral ICA-D on CTA	Carotid surgery, penetrating trauma, aortic dissection, common carotid dissection, or bilateral ICA-D	Yes	Yes	No	2	Yes	Patients with radiographically non dissected ICA	No

CTA, computed tomography angiography; FMD, fibromuscular dysplasia; ICA-D, internal carotid artery dissection; IS, ischemic stroke; MRI, magnetic resonance imaging; PA, pseudoaneurysm; TIA, transient ischemic attack.

* for the definition of the vessel wall.

**Table 2:** Detailed characteristics of the included patients and studies.

Study (Year)	Mean age (years) ± SD	Male *n* (%)	No cases/ controls	Cases SP-ICA distance, mm (mean ± SD)	Controls SP-ICA distance, mm (mean ± SD)	Cases SP length, mm (mean ± SD)	Controls SP length, mm (mean ± SD)	SP length measurement	Measurements of high-grade or complete occlusion of the ICA	SP-ICA distance measurement
Venturini et al. (2023) (9)	45 ± 7	42 (58)	31/41	5.9 ± 2.3	12.7 ± 1.6	NR	NR	Not performed	Not reported	Marginal SP-ICA distance of the contralateral side to the ICA-D was used for the analysis. Intercentric SP-ICA distance was measured ipsilateral to the ICA-D
Cruddas et al. (2021) (30)	47 ± 14	81 (54)	54/108	5.1 ± 14.0*	5.5 ± 25.0*	27.5 ± 22.0*	29.5 ± 44.0*	Performed in coronal view	Not reported	The closest distance on axial sections between the edge of the SP and the ICA
Amorim et al. (2018) (29)	50 ± 12	92 (70)	62/70	6.3 ± 1.9	7.1 ± 2.2	35.8 ± 14.4	30.4 ± 8.6	Performed on oblique MPR of CTA	Measurement of the center of the structure formed by the ICA lumen and the intramural hematoma	Measured between the edge of the SP and the ICA lumen in controls or the center of the structure formed by the ICA lumen and the intramural hematoma in cases
Renard et al. (2013) (28)	49 ± 10	102 (58)	88/88	6.8 ± 2.3	7.1 ± 2.5	NR	NR	Not performed	Measurement of the midpoint of the ICA lumen, as long as the reliable visualization of the ICA was possible	Margin of the SP and the supposed center of the ICA
Muthusami et al. (2013) (31)	54 ± 13	18 (17)	35/70	3.1 ± 1.8	5.0 ± 3.0	38.9 ± 7.4	36.4 ± 7.1	Maximum long axis of the SP, measured on oblique MPR	Not reported	The closest distance on axial sections between the edge of the SP and the ICA
Raser et al. (2011) (11)	51 ± 12	36 (47)	38/38	NR	5.2 ± 3.0	30.3 ± NR	26.2 ± 6.3	Determined by a Pythagorean calculation from measured rostral-caudal and axial values	Not reported	The closest distance on axial sections between the edge of the SP and the ICA

CCA-D, common carotid artery dissection; CTA, computed tomography angiography; ICA, internal carotid artery; ICA-D, internal carotid artery dissection; MPR, multiplanar reconstruction; NR, not reported; SD, standard deviation; SP, styloid process.

* SD values were derived from reported confidence intervals or interquartile ranges in the original study.

### Primary associations of interest

#### SP-ICA distance

Five studies were included in the primary meta-analysis. Two studies assessed intercentric SP-ICA distance (9, 29) and two studies reported marginal SP-ICA distance (30, 31). One study measured the distance between the center of the ICA lumen and the margin of the SP (28). The study by Venturini et al. measured the SP-ICA distance contralateral to the side of ICA-D (9). Across studies, SP–ICA distance was measured on axial CTA images; none used multiplanar reconstructions or true three-dimensional (3D) spatial distance measurements. Reporting of reliability was inconsistent. Muthusami et al. (31) reported modest interrater agreement for SP–ICA distance of 0.40, whereas Amorim et al. and Venturini et al. (9, 29) reported good interobserver agreement. A total of 270 patients and 377 controls were included.

**Figure 4:**
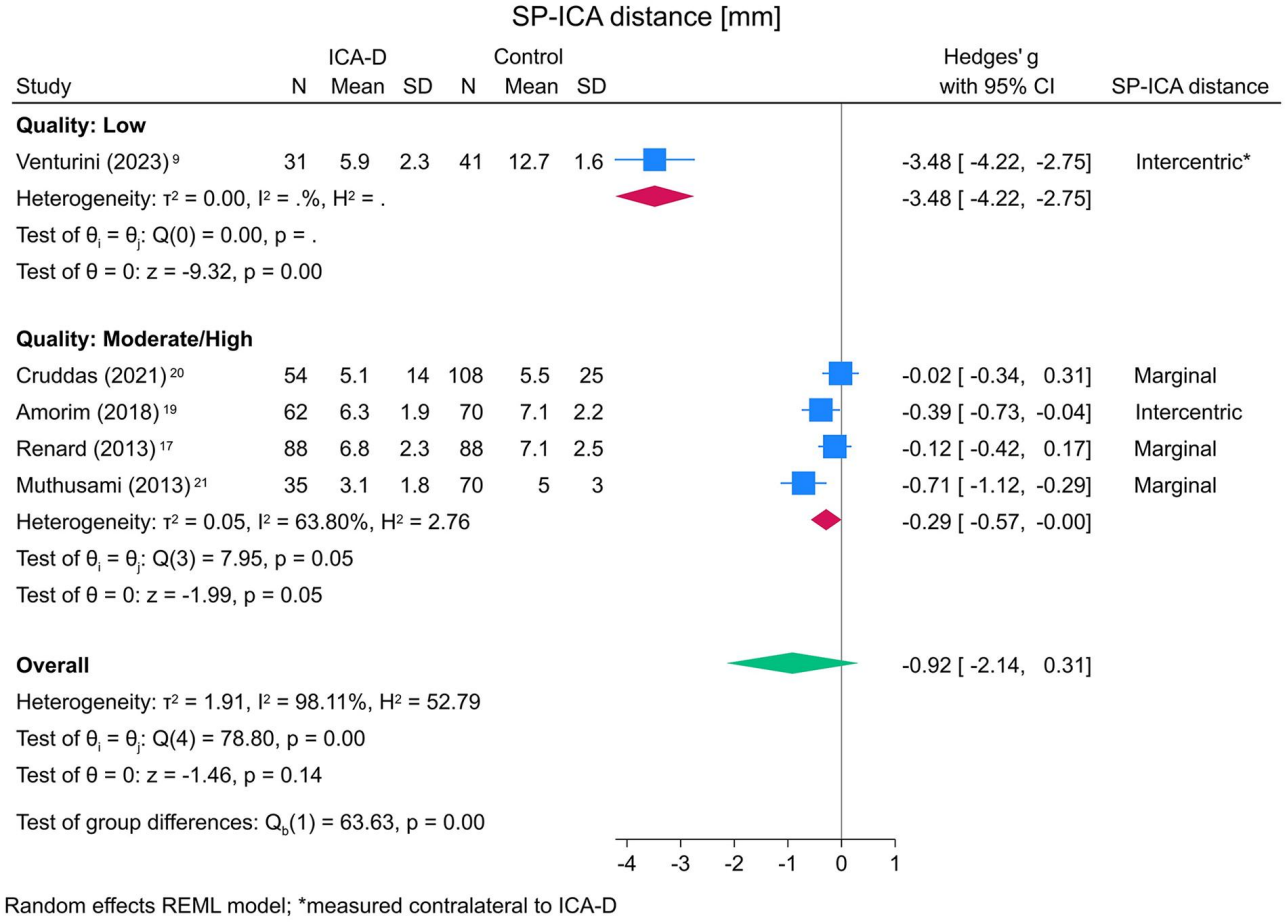
Forest plot of SP-ICA distance of five studies according to study quality (sorted by year of publication, descending). SP, styloid process; ICA, internal carotid artery; ICA-D, internal carotid artery dissection; N, number; SD, standard deviation; CI, confidence interval; REML, restricted maximum likelihood.

Figure 4 shows the meta-analysis of SP–ICA distance stratified by study quality. The single low-quality study by Venturini et al. (9) demonstrated a large negative effect size (SMD = −3.48 [−4.22, −2.75], *p* < 0.001) and was identified as highly influential in influence diagnostics, distorting the results. In contrast, the pooled analysis of the four moderate- to high-quality studies, measuring SP–ICA distance ipsilateral to ICA-D, showed a smaller but statistically significant effect (Hedges' g = −0.29 [−0.57, −0.00], *p* = 0.047), with moderate heterogeneity (I^2^ = 64%). When all studies were pooled regardless of study quality, the overall effect size was no longer statistically significant (SMD = −0.92 [−2.14, 0.31], *p* = 0.143) and was associated with substantial heterogeneity (I^2^ = 98%), largely reflecting the disproportionate influence of the low-quality study (9).

**Figure 5:**
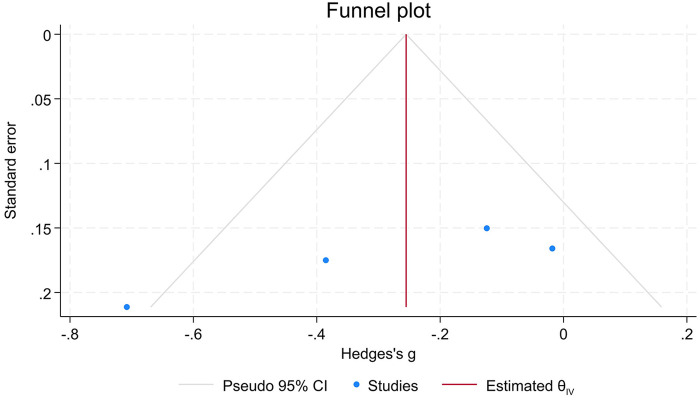
Funnel plot of the moderate/high-quality studies of the primary association.

No visual asymmetry was observed in the funnel plot on the four moderate/high-quality studies assessing SP-ICA distance (Figure 5). However, Egger's regression indicated a potential asymmetry (*p* = 0.013).

Two studies reported associations between SP-ICA distance and ICA-D using odds ratios, but results could not be pooled due to methodological differences. Amorim et al. (29) reported an OR of 0.77 (95% CI: 0.64–0.92) per 1 mm increase in intercentric SP-ICA distance (*p* = 0.004), indicating lower odds of ICA-D with increasing distance. Muthusami et al. (31) reported an OR of 7.58 (95% CI: 0.93–62.1; *p* = 0.060) for ICA-D in patients with a marginal SP-ICA distance <5 mm, suggesting higher risk with close SP–ICA proximity.

### Secondary associations of interest

#### Length of the styloid process

**Figure 6:**
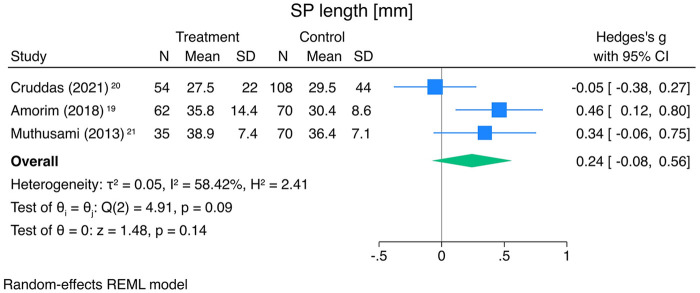
Forest plot SP length ipsilateral to the ICA-D (sorted by year of publication, descending). SP, styloid process; ICA-D, internal carotid artery dissection; N, number; SD, standard deviation; CI, confidence interval; REML, restricted maximum likelihood.

Three studies were included in the meta-analysis, comprising 151 ICA-D patients and 248 controls. No significant difference in SP length ipsilateral to the dissection was observed between groups (Figure 6; Hedges' g = 0.24 [−0.08, 0.56], *p* = 0.143). Two studies assessed the association using odds ratios with findings consistent with the meta-analysis reported by Abdelnour et al. (10). These results are not repeated here.

#### Angulation of the SP

Two studies assessed SP angulation. Muthusami et al. (31) found no significant difference in medial or anterior angulation between cases and controls (*p* = 0.640 and *p* = 0.180, respectively) Similarly, Raser et al. (11) reported no significant differences in medial angulation (*p* = 0.480) or caudal angulation (*p* = 0.320).

## Discussion

### Key findings and comparison with existing literature

Although SP is not widely recognized as a risk factor for ICA-D, emerging evidence suggests a potential association (32). SP length has been frequently reported in this context; however, given the anatomical variability of the SP, particularly in angulation and segmentation, SP length alone does not necessarily imply a close proximity to the ICA (11) and its role in ICA-D remains uncertain.

#### SP-ICA distance

Biomechanical considerations suggest that contact between the SP and the ICA may exert mechanical effects on the vessel wall (33). The pooled analysis including all studies showed substantial heterogeneity and should therefore be interpreted as exploratory, largely driven by a single low-quality and highly influential study. In contrast, the subgroup analysis restricted to moderate- and high-quality studies showed only moderate heterogeneity and a consistent direction of effect. Although this association reached conventional statistical significance, it should be interpreted cautiously and regarded as supportive rather than definitive evidence. Overall, these findings are consistent with the hypothesis that a shorter SP–ICA distance may be associated with ICA-D. Importantly, most studies did not describe measurement approaches in cases with high-grade stenosis or complete occlusion of the ICA, which may affect the reliability of SP–ICA distance assessment. Furthermore, CTA primarily assesses the vascular lumen and cannot reliably assess vessel wall pathology despite normal luminal appearance. MRI, particularly fat-saturated T1- and T2-weighted sequences, provides superior visualization of the vessel wall and is therefore more reliable in this context (34). Advanced techniques, such as high-resolution 3D black blood MRI, may further improve assessment of vessel wall pathology (35, 36). However, none of the included studies applied these methods.

A key limitation in SP-ICA distance measurement is the observed heterogeneity and relatively low interrater agreement, even within studies using identical measurement approaches. This variability likely reflects subjective selection of imaging slices and the lack of standardized anatomical reference points. For example, Muthusami et al. (31) reported a modest interrater agreement (0.40). Notably, all studies relied on axial CTA images, without the use of multiplanar reconstructions (MPR) or 3D measurement techniques. Given the complex 3D relationship between the SP and the cervical ICA, reliance on axial planes alone may underestimate the true minimum distance and contribute to measurement variability. Standardized and reproducible measurement protocols across imaging modalities are therefore essential for future research.

Only a limited number of studies have assessed intercentric SP–ICA distance, and interpretation is further complicated by the lack of stable reference points due to variations in ICA-D presentations, such as stenosis or pseudoaneurysm, as well as by head and neck positioning during imaging (9, 29). Dynamic imaging, such as 3D simulation, suggests that ICA impingement may occur in flexion or rotation (37). Emerging evidence supports the use of positional CTA protocols, including imaging during head movement, to improve detection of SP–ICA interactions (38–40).

#### SP length

Although the SP length has been evaluated in a previous meta-analysis (10), methodological limitations warrant cautious interpretation. These include a limited number of screened studies and incomplete reporting of key statistical data, such as standard deviation data (11), potentially compromizing the robustness of pooled estimates. Moreover, SP length alone may not adequately reflect the anatomical relationship between the SP and the ICA. Consistent with our findings, no significant association between SP length and ICA-D was observed, suggesting that distance-based metrics may be more informative.

#### Other morphological styloid characteristics

Based on anatomical considerations, a more medial and anterior angulation of the SP could reduce the distance to the ICA. However, only two observational studies assessed SP angulation using different measurement approaches (11, 31), and neither demonstrated a significant association with ICA-D. Other morphological features, such as SP thickness or segmentation, have not been systematically evaluated. Further studies incorporating these characteristics may provide a more comprehensive understanding of the role of SP morphology in ICA-D.

### Deviations of the protocol

Beyond SP length and SP-ICA distance, the meta-analysis was limited by the availability and heterogeneity of data on additional SP characteristics. For example, SP segmentation was not evaluated in any included study, and measurement methods for SP angulation varied substantially. Furthermore, the SP-ICA distance required subclassification into intercentric and marginal measurements due to methodological differences. In one study (28), SP-ICA distance was classified as intercentric despite being measured to the margin of the SP, highlighting inconsistencies in measurement definitions.

### Strengths and contributions

This study has several strengths. The systematic and transparent methodology was developed in collaboration with a medical information specialist and implemented by a multidisciplinary team, thereby minimizing the risk of bias. Independent screening by multiple reviewers, supervised by a board-certified neuroradiologist, enhanced the accuracy of study selection and data extraction. Adherence to established systematic review standards supports the robustness of the findings and provides a comprehensive synthesis of the current evidence while clearly delineating its limitations.

### Future directions

Current evidence on SP–ICA relationships remains limited and heterogeneous. Given the anatomical variability of both the SP and the ICA, SP–ICA distance appears to be a more relevant parameter than SP length alone (41, 42). Future studies should employ standardized and reproducible measurement protocols and incorporate advanced imaging techniques, particularly high-resolution MRI for vessel wall assessment.

CTA performed during the acute phase of dissection may be limited by luminal stenosis and intramural hematoma. MRI, especially with fat-suppressed sequences, remains the preferred modality for detailed vessel wall evaluation. Although ultrasound may offer additional insights, its operator dependency and limited reliability in acute emergency settings restrict its routine use. Large-scale prospective studies integrating imaging, clinical risk factors, and potential genetic predispositions are needed to better define the role of SP morphology in ICA-D.

### Limitations

Several limitations should be considered. The available evidence is based exclusively on retrospective observational studies, limiting causal inference. Despite efforts to improve comparability, substantial heterogeneity in SP-ICA distance measurement techniques remain. In particular, all included studies have relied on axial CTA images without the use of multiplanar or three-dimensional reconstruction methods, which may not accurately capture the true shortest distance and may contribute to measurement variability. Quantitative risk estimation was not feasible due to limited data and methodological heterogeneity. In addition, inconsistent exclusion of traumatic ICA-D across studies may have introduced confounding bias. Overall, this meta-analysis cannot establish a causal relationship between SP characteristics and ICA-D.

### Implications for practice and research

Standardized imaging protocols and measurement techniques are essential for advancing research in this field. Defining clinically relevant thresholds for SP–ICA distance may improve risk stratification. Interdisciplinary collaboration among neuroradiologists, neurologists, and ENT specialists will be important to guide diagnosis and management. In selected cases, surgical interventions such as styloidectomy may be considered as part of secondary prevention strategies. Ultimately, improved understanding of SP–ICA interactions may contribute to reducing recurrence of ICA-D and improving long-term outcomes.

## Conclusion

Despite heterogeneity in measurement techniques, ICA-D appears to be associated with a shorter SP-ICA distance. In contrast, SP length does not differ significantly between patients with and without ICA-D. Future research should focus on standardized imaging methodologies and prospective study designs to clarify the clinical relevance of SP–ICA relationships and to inform evidence-based management strategies.

## Data Availability

The raw data supporting the conclusions of this article will be made available by the authors, without undue reservation.

## References

[1] Blum CA Yaghi S. Cervical artery dissection: a review of the epidemiology, pathophysiology, treatment, and outcome. Arch Neurosci. (2015) 2:e26670. 10.5812/archneurosci.26670 26478890 PMC4604565

[2] Rubinstein SM Peerdeman SM van Tulder MW Riphagen I Haldeman S. A systematic review of the risk factors for cervical artery dissection. Stroke. (2005) 36:1575–80. 10.1161/01.STR.0000169919.73219.30 15933263

[3] Gunduz ME Kadirvel R Kallmes DF Pezzini A Keser Z. Spontaneous cervical artery dissection: is it really a connective tissue disease? A comprehensive review. Front Neurol. (2023) 14:1241084. 10.3389/fneur.2023.1241084 37885478 PMC10598645

[4] Galyfos G Filis K Sigala F Sianou A. Traumatic carotid artery dissection: a different entity without specific guidelines. Vasc Specialist Int. (2016) 32:1–5. 10.5758/vsi.2016.32.1.1 27051653 PMC4816018

[5] Patel RR Adam R Maldjian C Lincoln CM Yuen A Arneja A. Cervical carotid artery dissection: current review of diagnosis and treatment. Cardiol Rev. (2012) 20:145–52. 10.1097/CRD.0b013e318247cd15 22301716

[6] Engelter ST Grond-Ginsbach C Metso TM Metso AJ Kloss M Debette S Cervical artery dissection: trauma and other potential mechanical trigger events. Neurology. (2013) 80:1950–7. 10.1212/WNL.0b013e318293e2eb 23635964

[7] Tadjer J Béjot Y. Vascular variant of eagle syndrome: a review. Front Neurol. (2024) 15:1463275. 10.3389/fneur.2024.1463275 39440251 PMC11493652

[8] Keser Z Chiang C-C Benson JC Pezzini A Lanzino G. Cervical artery dissections: etiopathogenesis and management. Vasc Health Risk Manag. (2022) 18:685–700. 10.2147/VHRM.S362844 36082197 PMC9447449

[9] Venturini G Vuolo L Pracucci G Picchioni A Failli Y Benvenuti F The proximity between styloid process and internal carotid artery as a possible risk factor for dissection: a case–control study. Neuroradiology. (2023) 65:915–22. 10.1007/s00234-023-03121-0 36750496 PMC10105656

[10] Abdelnour LH Kurdy M Idris A. Association of styloid process length with cervical carotid artery dissection: meta-analysis. Health Sci Rev. (2023) 9:100134. 10.1016/j.hsr.2023.100134

[11] Raser JM Mullen MT Kasner SE Cucchiara BL Messé SR. Cervical carotid artery dissection is associated with styloid process length. Neurology. (2011) 77:2061–6. 10.1212/WNL.0b013e31823b4729 22116948

[12] Page MJ McKenzie JE Bossuyt PM Boutron I Hoffmann TC Mulrow CD The PRISMA 2020 statement: an updated guideline for reporting systematic reviews. Br Med J. (2021) 372:n71. 10.1136/bmj.n71 33782057 PMC8005924

[13] Klail T Kalioras E von Gernler M Müller M Wagner F. Specific anatomical characteristics of the styloid process as risk factors for internal carotid artery dissections: a systematic review and meta-analysis of controlled trials. PROSPERO (2024). Available online at: https://www.crd.york.ac.uk/prospero/display_record.php?ID=CRD42024582594 (Accessed February 16, 2025) 10.3389/fradi.2026.1788985 PMC13191739 42182935

[14] Badhey A Jategaonkar A Anglin Kovacs AJ Kadakia S De Deyn PP Ducic Y Eagle syndrome: a comprehensive review. Clin Neurol Neurosurg. (2017) 159:34–8. 10.1016/j.clineuro.2017.04.021 28527976

[15] Veritas Health Innovation. Covidence systematic review software (2026). Available online at: https://www.covidence.org (Accessed January 23, 2026)

[16] Oppenheim C Naggara O Touzé E Lacour J-C Schmitt E Bonneville F High-resolution MR imaging of the cervical arterial wall: what the radiologist needs to know. RadioGraphics. (2009) 29:1413–31. 10.1148/rg.295085183 19755603

[17] Wells GA Shea B O’Connell D Peterson J Welch V Losos M Tugwell P. The Newcastle-Ottawa scale (NOS) for assessing the quality of non-randomised studies in meta-analysis. Ottawa Hospital Research Institute (2026). Available online at: https://www.ohri.ca/programs/clinical_epidemiology/oxford.asp (Accessed January 23, 2026)

[18] Higgins JPT Thomas J Chandler J Cumpston M Li T Page MJ Cochrane Handbook for Systematic Reviews of Interventions. London: Cochrane (2024).

[19] Tukey JW. Exploratory Data Analysis. Reading. Reading, MA: Addison-Wesley (1977).

[20] Mantovani G Zangrossi P Flacco ME Di Domenico G Nastro Siniscalchi E De Ponte FS Styloid jugular nutcracker: the possible role of the styloid process spatial orientation—a preliminary morphometric computed study. Diagnostics. (2023) 13:298. 10.3390/diagnostics13020298 36673108 PMC9857444

[21] Shah SP Praveen N Syed V Subhashini A. Elongated styloid process: a retrospective panoramic radiographic study. World J Dent. (2012) 3:316–9. 10.5005/jp-journals-10015-1181

[22] Gervickas A Kubilius R. Peculiarities of the investigation, clinics and treatment of stylohyoid syndrome and glossopharyngeal neuropathy. Medicina (Kaunas). (2004) 40:943–8. 15516816

[23] Báez-Martínez EM Blesa LM Guijarro BS Gonzalez CO Vinagre IN García Torres MA. Eagle syndrome. Pract Neurol. (2021) 21:548–9. 10.1136/practneurol-2021-002949 34389645

[24] Colby CC Del Gaudio JM. Stylohyoid complex syndrome. Arch Otolaryngol Head Neck Surg. (2011) 137:248. 10.1001/archoto.2011.25 21422308

[25] Tardivo V Castaldi A Baldino G Siri G Bruzzo M Del Sette M Internal carotid artery dissection related to abnormalities of styloid process: is it only a matter of length? Neurol Sci. (2022) 43:459–65. 10.1007/s10072-021-05350-8 34059959

[26] Tapia DQ Castellaccio A Arrojo FG Munoz AH Viano J Anciones B Acute cervical artery dissections beware of your neck!. Neuroradiology. (2017) 59:1–115.

[27] Shah S Sen S Mascari R King J Gambrell A. Abstract 1122-000020: assessing correlation between styloid length and internal carotid artery dissection in eagle syndrome patients. Stroke Vasc Interv Neurol. (2021) 1:e12221. 10.1161/SVIN.01.suppl_1.000020

[28] Renard D Azakri S Arquizan C Swinnen B Labauge P Thijs V. Styloid and hyoid bone proximity is a risk factor for cervical carotid artery dissection. Stroke. (2013) 44:2475–9. 10.1161/STROKEAHA.113.001444 23908063

[29] Amorim JM Pereira D Rodrigues MG Beato-Coelho J Lopes M Cunha A Anatomical characteristics of the styloid process in internal carotid artery dissection: case–control study. Int J Stroke. (2018) 13:400–5. 10.1177/1747493017730779 28906206

[30] Cruddas L Joffe M Baker D. Can styloid process and internal carotid artery anatomy be used to predict carotid artery dissection? Ann Vasc Surg. (2021) 74:105–10. 10.1016/j.avsg.2020.12.034 33549788

[31] Muthusami P Kesavadas C Sylaja PN Thomas B Harsha KJ Kapilamoorthy TR. Implicating the long styloid process in cervical carotid artery dissection. Neuroradiology. (2013) 55:861–7. 10.1007/s00234-013-1186-1 23579551

[32] Klail T Sachs L Panos LD Urban OY Siller T Pilgram-Pastor S Styloid process-related internal carotid artery dissection: extensive literature review of diagnosis, treatment and outcomes (exemplified with a single-center case series). Neuroradiology. (2025) 67:1355–64. 10.1007/s00234-025-03616-y 40285871 PMC12357798

[33] Horio Y Fukuda K Miki K Hirao N Iwaasa M Abe H Dynamic assessment of internal carotid artery and elongated styloid process in a case of bilateral carotid artery dissection. Surg Neurol Int. (2020) 11:163. 10.25259/SNI_42_2020 32637216 PMC7332696

[34] Mehdi E Aralasmak A Toprak H Yildiz S Kurtcan S Kolukisa M Craniocervical dissections: radiologic findings, pitfalls, mimicking diseases: a pictorial review. Curr Med Imaging Rev. (2018) 14:207–22. 10.2174/1573405613666170403102235 29853818 PMC5902863

[35] Zhu X Qiu H Hui FK Zhang Y Liu Y Man F Practical value of three-dimensional high resolution magnetic resonance vessel wall imaging in identifying suspicious intracranial vertebrobasilar dissecting aneurysms. BMC Neurol. (2020) 20:199. 10.1186/s12883-020-01779-0 32434485 PMC7238595

[36] Shi Z Tian X Tian B Meddings Z Zhang X Li J Identification of high risk clinical and imaging features for intracranial artery dissection using high-resolution cardiovascular magnetic resonance. J Cardiovasc Magn Reson. (2021) 23:74. 10.1186/s12968-021-00766-9 34120627 PMC8201847

[37] Nastro Siniscalchi E Mormina E Cicchiello S Granata F Vinci SL Galletta K Can a 3D virtual imaging model predict eagle syndrome? Appl Sci. (2022) 12:4564. 10.3390/app12094564

[38] Nastro Siniscalchi E Raffa G Germanò A De Ponte FS Galletti F. Regarding chronic cerebrospinal venous insufficiency and meniere’s disease: interventional versus medical therapy. Laryngoscope. (2021) 131:E980. 10.1002/lary.28870 32579709

[39] Nastro Siniscalchi E Raffa G Vinci S Granata F Pitrone A Tessitore A Eagle syndrome: lights and shadows of an underestimated condition of multidisciplinar interest. Adv Oral Maxillofac Surg. (2022) 5:100243. 10.1016/j.adoms.2021.100243

[40] Brassart N Deforche M Goutte A Wery D. A rare vascular complication of eagle syndrome highlight by CTA with neck flexion. Radiol Case Rep. (2020) 15:1408–12. 10.1016/j.radcr.2020.05.052 32636984 PMC7330452

[41] Di Pino L Franchina AG Costa S Gangi S Strano F Ragusa M Prevalence and morphological changes of carotid kinking and coiling in growth: an echo-color Doppler study of 2856 subjects between aged 0 to 96 years. Int J Cardiovasc Imaging. (2021) 37:479–84. 10.1007/s10554-020-02014-0 32914402 PMC7900048

[42] Langlais RP Miles DA Van Dis ML. Elongated and mineralized stylohyoid ligament complex: a proposed classification and report of a case of eagle’s syndrome. Oral Surg Oral Med Oral Pathol Oral Radiol. (1986) 61:527–32. 10.1016/0030-4220(86)90400-7 3459129

