# Supplementary material for: Anatomical characteristics of the styloid process associated with internal carotid artery dissection: a systematic review and meta-analysis of controlled trials
Source: Front Radiol. 2026 May 1;6:1788985. doi: 10.3389/fradi.2026.1788985 (PMC13191739; doi:10.3389/fradi.2026.1788985)
Supplement: Supplementary file 1 [file Supplementaryfile1.docx]

# **SUPPLEMENTARY FILES**

**-**

**Specific anatomical characteristics of the styloid process as risk factors for internal carotid artery dissections: a systematic review and meta-analysis of controlled trials**

## **Supplementary methods 1 – Full search strategies for different databases**

**Database: Ovid MEDLINE(R) ALL (1946 -- 10/07/2024)**

Search date: 11/07/2024

Reference: von Gernler,Marc and Klail,Tomas and Müller,Martin, searchRxiv.2025.00853, searchRxiv, doi:10.1079/searchRxiv.2025.00853, CABI, Specific anatomical characteristics of the styloid process as risk factors for internal carotid artery dissections [Ovid MEDLINE]., (2025)

Search Strategy:

----------------------------------------------------------------------

| ID | Search terms | Hits |
| --- | --- | --- |
| 1 | Temporal Bone/ | 12471 |
| 2 | (styloid* or stylohyoid* or stylocarotid* or stylo-hyoid* or stylo-carotid*).ti,ab,kf. | 2832 |
| 3 | eagle*.ti,ab,kf. | 8268 |
| 4 | or/1-3 | 22295 |
| 5 | carotid arteries/ or carotid artery, common/ or carotid artery, internal/ or carotid sinus/ | 62098 |
| 6 | Carotid Artery, Internal, Dissection/ or Dissection, Blood Vessel/ | 1491 |
| 7 | carotid artery injuries/ or carotid artery, internal, dissection/ | 5401 |
| 8 | ((caroti* or cervic*) adj3 (arter* or ruptur* or pseudoaneur* or pseudo-aneur* or dissect* or injur* or lesion* or false aneur* or tear? or damage* or lacerat* or trauma*)).ti,ab,kf. | 116445 |
| 9 | (ICA-D or ICAD or CCAD or CAD or ICA or ACI).ti,ab,kf. | 79320 |
| 10 | or/5-9 | 212619 |
| 11 | and/4,10 | 662 |

----------------------------------------------------------------------

**Database: Ovid Embase (1974 -- 28/07/2023)**

Search date: 31/07/2023

Reference: von Gernler,Marc and Klail,Tomas and Müller,Martin, searchRxiv.2025.00852, searchRxiv, doi:10.1079/searchRxiv.2025.00852, CABI, Specific anatomical characteristics of the styloid process as risk factors for internal carotid artery dissections [Ovid Embase]., (2025)

Search Strategy:

----------------------------------------------------------------------

| ID | Search terms | Hits |
| --- | --- | --- |
| 1 | temporal bone/ | 15502 |
| 2 | styloid process*.dq. | 238 |
| 3 | (styloid* or stylohyoid* or stylocarotid* or stylo-hyoid* or stylo-carotid*).ti,ab,kf. | 3217 |
| 4 | eagle*.ti,ab,kf. | 9780 |
| 5 | or/1-4 | 27530 |
| 6 | exp carotid artery/ | 140338 |
| 7 | carotid artery injury/ | 5828 |
| 8 | artery dissection/ | 11243 |
| 9 | ((caroti* or cervic*) adj3 (arter* or ruptur* or pseudoaneur* or pseudo-aneur* or dissect* or injur* or lesion* or false aneur* or tear? or damage* or lacerat* or trauma*)).ti,ab,kf. | 157485 |
| 10 | (ICA-D or ICAD or CCAD or CAD or ICA or ACI).ti,ab,kf. | 129421 |
| 11 | or/6-10 | 351607 |
| 12 | and/5,11 | 1373 |

----------------------------------------------------------------------

**Database: CINAHL with Full Text (1963 -- Present)**

Interface: EBSCOhost Research Databases

Search Screen: Advanced Search

Expanders: Apply equivalent subjects

Search modes: Find all my search terms

Search date: 11/07/2024

Reference: von Gernler,Marc and Klail,Tomas and Müller,Martin, searchRxiv.2025.00850, searchRxiv, doi:10.1079/searchRxiv.2025.00850, CABI, Specific anatomical characteristics of the styloid process as risk factors for internal carotid artery dissections [CINAHL]., (2025)

Search Strategy:

----------------------------------------------------------------------

| ID | Search terms | Hits |
| --- | --- | --- |
| S1 | (MH "Eagle Syndrome") | 54 |
| S2 | (MH "Temporal Bone") | 2653 |
| S3 | TX (stylo* OR eagle*) | 9989 |
| S4 | S1 OR S2 OR S3 | 12496 |
| S5 | (MH "Carotid Arteries") OR (MH "Carotid Artery Diseases") OR (MH "Carotid Artery Dissections") | 12062 |
| S6 | TI ((caroti* OR cervic*) N3 (arter* OR ruptur* OR pseudoaneur* OR pseudo-aneur* OR dissect* OR injur* OR lesion* OR "false aneur*" OR tear# OR damage* OR lacerat* OR trauma*)) OR AB ((caroti* OR cervic*) N3 (arter* OR ruptur* OR pseudoaneur* OR pseudo-aneur* OR dissect* OR injur* OR lesion* OR "false aneur*" OR tear# OR damage* OR lacerat* OR trauma*)) | 21158 |
| S7 | TI ( (ICA OR ACI OR ICA-D OR ICAD OR CCAD OR CAD) ) OR AB ( (ICA OR ACI OR ICA-D OR ICAD OR CCAD OR CAD) ) | 15459 |
| S8 | S5 OR S6 OR S7 | 39919 |
| S9 | S4 AND S8 | 179 |

----------------------------------------------------------------------

**Database: Cochrane Library (1996 – Present)**

Search date: 11/07/2024

Reference: von Gernler,Marc and Klail,Tomas and Müller,Martin, searchRxiv.2025.00851, searchRxiv, doi:10.1079/searchRxiv.2025.00851, CABI, Specific anatomical characteristics of the styloid process as risk factors for internal carotid artery dissections [Cochrane Library]., (2025)

Search Strategy:

----------------------------------------------------------------------

| ID | Search terms | Hits |
| --- | --- | --- |
| #1 | [mh ^"Temporal Bone"] | 59 |
| #2 | (styloid* OR stylohyoid* OR stylocarotid* OR stylo-hyoid* OR stylo-carotid*):ti,ab,kw | 185 |
| #3 | eagle*:ti,ab,kw | 197 |
| #4 | {OR #1-#3} | 431 |
| #5 | [mh ^"carotid arteries"] OR [mh ^"carotid artery, common"] OR [mh ^"carotid artery, internal"] OR [mh ^"carotid sinus"] | 1512 |
| #6 | [mh ^"Carotid Artery, Internal, Dissection"] OR [mh ^"Dissection, Blood Vessel"] | 10 |
| #7 | [mh ^"carotid artery injuries"] OR [mh ^"carotid artery, internal, dissection"] | 28 |
| #8 | ((caroti* OR cervic*) NEAR/3 (arter* OR ruptur* OR pseudoaneur* OR pseudo-aneur* OR dissect* OR injur* OR lesion* OR ("false" NEAR/2 aneur*) OR tear? OR damage* OR lacerat* OR trauma*)):ti,ab,kw | 8997 |
| #9 | (ICA-D OR ICAD OR CCAD OR CAD OR ICA OR ACI):ti,ab,kw | 7905 |
| #10 | {OR #5-#9} | 16505 |
| #11 | {AND #4, #10} | 5 |

**Database: Scopus (1970 – Present)**

Search date: 11/07/2024

Search results: 453

Reference: von Gernler,Marc and Klail,Tomas and Müller,Martin, searchRxiv.2025.00854, searchRxiv, doi:10.1079/searchRxiv.2025.00854, CABI, Specific anatomical characteristics of the styloid process as risk factors for internal carotid artery dissections [Scopus]., (2025)

Search Strategy:

----------------------------------------------------------------------

(TITLE-ABS-KEY(("styloid*") OR ("stylohyoid*") OR ("stylocarotid*") OR ("stylo-hyoid*") OR ("stylo-carotid*") OR ("eagle*")))

AND

(TITLE-ABS-KEY(( "caroti*" OR "cervic*" ) W/2 ( "arter*" OR "ruptur*" OR "pseudoaneur*" OR "pseudo-aneur*" OR "dissect*" OR "injur*" OR "lesion*" OR "false aneur*" OR "tear?" OR "damage*" OR "lacerat*" OR "trauma*" OR ICA-D OR ICAD OR CCAD OR CAD OR ICA OR ACI)))

----------------------------------------------------------------------

**Database: Web of Science Core Collection**

Entitlements:

- WOS.SCI: 1900 to 2024

- WOS.AHCI: 1975 to 2024

- WOS.ESCI: 2019 to 2024

- WOS.ISTP: 1990 to 2024

- WOS.SSCI: 1900 to 2024

- WOS.ISSHP: 1990 to 2024

Search date: 11/07/2024

Reference: von Gernler,Marc and Klail,Tomas and Müller,Martin, searchRxiv.2025.00855, searchRxiv, doi:10.1079/searchRxiv.2025.00855, CABI, Specific anatomical characteristics of the styloid process as risk factors for internal carotid artery dissections [Web of Science Core Collection]., (2025)

Search Strategy:

----------------------------------------------------------------------

| ID | Search terms | Hits |
| --- | --- | --- |
| 1 | TS=("styloid*" OR "stylohyoid*" OR "stylocarotid*" OR "stylo-hyoid*" OR "stylo-carotid*" OR "eagle*") | 20359 |
| 2 | TS=(( "caroti*" OR "cervic*" ) NEAR/2 ( "arter*" OR "ruptur*" OR "pseudoaneur*" OR "pseudo-aneur*" OR "dissect*" OR "injur*" OR "lesion*" OR "false aneur*" OR "tear*" OR "damage*" OR "lacerat*" OR "trauma*" )) | 118523 |
| 3 | TS=(ICA-D or ICAD or CCAD or CAD or ICA or ACI) | 141317 |
| 4 | #2 OR #3 | 252191 |
| 5 | #4 AND #1 | 283 |

----------------------------------------------------------------------

## **Supplementary table 1 – Individual reasons for exclusion of the studies after full-text assessment**

| Study | Year | Reason for exclusion |
| --- | --- | --- |
| Mantovani et al. | 2023 | Wrong study focus - Lack of ICA-D assessment |
| Tardivo el al. | 2022 | Wrong study design - No healthy controls, nut the contralateral side of cases |
| Abdelnour et al. | 2023 | Wrong study design - Meta-analysis |
| Shah et al. | 2012 | Wrong study focus - Lack of ICA-D assessment |
| Gervickas et al. | 2004 | Wrong study focus - Lack of ICA-D assessment |
| Baez-Martinez et al. | 2021 | Wrong study focus - Lack of ICA-D assessment |
| Tapia et al. | 2017 | Conference paper |
| Colby et al. | 2011 | Wrong study focus - Lack of ICA-D assessment |
| Shah et al. | 2021 | Abstract |

## **Supplementary table 2 – Individual study NOS scoring**

| Newcastle scoring | Venturini et al. | Cruddas et al. | Amorim et al. | Renard et al. | Muthusami et al. | Raser et al. |
| --- | --- | --- | --- | --- | --- | --- |
| Case definition adequate |  |  |  |  | * |  |
| Representativeness of the cases |  | * | * | * | * | * |
| Selection of controls |  |  |  |  |  |  |
| Definition of controls | * | * | * | * | * | * |
| Main factor | * | * | * | * | * | * |
| Additional factor | * | * | * | * | * | * |
| Ascertainment of exposure | * | * | * | * | * | * |
| Same method of ascertainment for cases and controls | * | * | * | * | * | * |
| Non-response rate | * | * | * | * | * | * |
| Score | 6 | 7 | 7 | 7 | 8 | 7 |
